# Controlling HIV sexual transmission: a major challenge for China’s new leadership

**DOI:** 10.1186/2045-3701-3-17

**Published:** 2013-03-19

**Authors:** Shibo Jiang, Weihua Li, Lu Lu

**Affiliations:** 1 Key Laboratory of Medical Molecular Virology of Ministries of Education and Health, Shanghai Medical College, Fudan University, Shanghai, China; 2 Shanghai Institute of Planned Parenthood Research, WHO Collaborating Center for Research in Human Reproduction, Shanghai, China

## Abstract

China’s new leadership has been on board recently and they will face a great challenge, how to control the spread of HIVAIDS in China. Recent studies have shown that sexual transmission has become the main route of HIV spread in China. Therefore, more strong and effective measures have to be taken to protect people from HIV infection via sexual transmission in order to reduce the mortality and morbidity of HIV infection and AIDS in China.

## 

During the week of World AIDS Day 2012, Mr. Li Keqiang, Chinese Vice-Premier and key member of China’s new leadership, warned that controlling the spread of HIV/AIDS remains a serious public health problem, stating that it is “not only a medical issue, but also a social challenge.” Under these circumstances, control over this epidemic requires cooperation between both government groups and non-governmental organizations (http://news.xinhuanet.com/english/bilingual/2012-11/29/c_132007520.htm).

By the end of October 2012, a total of 492,191 HIV/AIDS cases had been reported on the mainland of China, even though the government’s estimation is much higher at 780,000. While, nationally, the prevalence of HIV/AIDS remains low, the epidemic has become severe in some areas and among some populations [1].

The Chinese government has implemented much stronger commitment to HIV/AIDS prevention since 2003 by accelerating strategic planning and scaling-up the initiatives to conduct HIV testing and control the epidemic in high-risk groups, including injection drug users, sex workers, men who have sex with men (MSM), and plasma donors, as well as providing treatment for infected individuals [2]. As a result, a significant reduction of the incidence of HIV transmission has occurred among injection drug users and blood donors. Also, the rate of mother-to-child transmission of HIV has plunged from 34.8% in 2007 to 7.4% in 2012 (http://news.xinhuanet.com/english/bilingual/2012-11/29/c_132007520.htm).

Conversely, however, a more serious problem has arisen in recent years. Namely, the proportion of reported HIV/AIDS cases involving homosexual and heterosexual transmission has climbed successively each year since 2004 (Figure 1) [3]. For example, the reported cases resulting from HIV sexual transmission increased from 33.1% in 2006 to 76.3% in 2011 [1]. From January to October of 2012, China reported 68,802 new HIV/AIDS cases, and about 85% of them represented infection through sexual contact, including 21% through MSM. Therefore, sexual transmission has become the main route of HIV spread in China [1].

**Figure 1:**
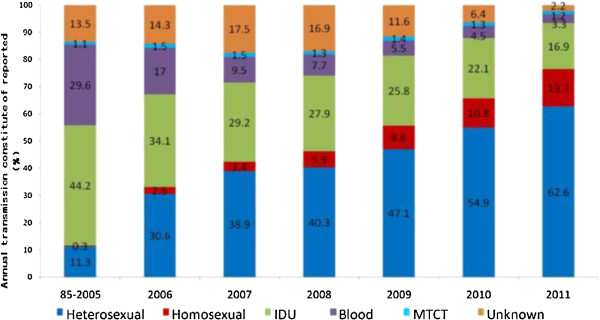
**Breakdown of the newly reported HIV/AIDS cases by year and transmission route [adapted from [**[1]**]].**

An even more ominous sign is the current spread from high-risk populations to the general population [3], especially the married women. Thus, greater attention should be paid to those women, in addition to the high-risk populations. A survey indicates that 30.5% of males soliciting sex with female prostitutes consistently use condoms, while consistent condom usage with their spouses is only 3.5% [4]. Most married women have established a degree of trust which seems to obviate the need to take preventive measures when engaging in sexual intercourse with their husbands. However, some of their spouses may also have had sex with prostitutes, and, as a consequence, may pass on the viruses.

Besides condom use, other effective means, including public education and intervention, are also important for preventing HIV sexual transmission. A number of Chinese central government departments have implemented information, education and communication (IEC) plans. As a results, some success in educating the public has been demonstrated, as indicated by a 2010 study showing that the levels of basic awareness of HIV among young students and male migrants reached 88.2% and 75.3%, respectively [1]. Overall, the Chinese government, in collaboration with disease control bodies, medical treatment facilities and community-based organizations, has strengthened its intervention program among the high-risk populations, especially among prostitutes and MSM, in order to reduce the sexual transmission of HIV. Data from the national sentinel surveillance indicate that intervention among sex workers increased from 74.3% in 2009 to 81.0% in 2011, and among MSM, it increased from 75.1% in 2009 to 76.7% in 2011 [1].

The treatment-as-prevention is a very promising approach against HIV sexual transmission. Most recently, Jia et al compared the annual rate of HIV infection in HIV-negative individuals who had sex with treated or treatment-naive HIV-positive partners. They found that the antiretroviral treatment resulted in an overall 26% reduction in HIV transmission, suggesting that antiretroviral therapy prevents HIV transmission among heterosexual, serodiscordant couples [5].

Microbicides are antimicrobial medications formulated as a gel, cream, suppository, or intravaginal ring for vaginal (or rectal) administration, to prevent the sexual transmission of HIV [6]. A clinical trial conducted in South Africa has shown that a microbicide gel containing 1% Tenofovir, an anti-HIV drug, is 39% effective at preventing sexual transmission of HIV to women during sex with HIV-positive male partners [7]. It is expected that such microbicides will play an increasingly critical role in the prevention of HIV sexual transmission in the near future. However, development of anti-HIV microbicides has been remarkably neglected in China. Before 2008, no major funding supported microbicide development in China. In 2009, four projects did receive funding from the Ministry of Health and Ministry of Science and Technology of P. R. China. However, the total budget for 2010 and 2011 was only 3.6 M USD, while the U.S. National Institutes of Health provided 147 M USD to support microbicide development for the same period (http://www.hivresourcetracking.org).

So far, no safe and effective microbicide has been approved for clinical use in the world. However, in China, several so-called “microbicides” have been on the market for several years. These “microbicides” contain surfactants, such as nonoxynol-9 (N-9), octoxynol-9 (also known as Triton X-100) or benzalkonium chloride (BZK). Although these chemicals are effective in killing viruses *in vitro*, they can be toxic to the vaginal mucosa *in vivo*. Clinical trials have shown that N-9 does not reduce HIV risk, but actually increases it [8]. We pointed out this problem in 2006 [9], but we have seen no progress in solving it.

HIV/AIDS studies have historically been hampered by an overly cautious biosafety policy executed in China, which requires that samples containing live HIV must be handled in a BSL-3 facility (http://www.lncdc.com/fgbz/gzfa/rjcybyml.doc). However, in the United States and many other countries, the experiments using research-laboratory-scale quantities of HIV, like HBV and other blood-borne viruses, can be performed in a BSL-2 facility (http://www.cdc.gov/biosafety/publications/bmbl5), because HIV is not a dangerous airborne virus that must be handled in BSL-3 facilities. Because of a limited number of BSL-3 facilities located in a few big cities and because of the high cost of using a BSL-3 facility, most HIV/AIDS researchers cannot carry out their key experiments using live HIV virions. Consequently, if live HIV is needed, a number of research projects must be discontinued. Therefore, we suggest that this policy be revised so that the researchers in China can handle research-laboratory-scale quantities of HIV in BSL-2 facilities as described by the US CDC (http://www.cdc.gov/biosafety/publications/bmbl5).

## Competing interest

The authors declare that they have no conflicts of interest.
